# Mechanically-flexible wafer-scale integrated-photonics fabrication platform

**DOI:** 10.1038/s41598-024-61055-w

**Published:** 2024-05-09

**Authors:** Milica Notaros, Thomas Dyer, Andres Garcia Coleto, Ashton Hattori, Kevin Fealey, Seth Kruger, Jelena Notaros

**Affiliations:** 1 Research Laboratory of Electronics, Massachusetts Institute of Technology, Cambridge, MA 02139 USA; 2 New York Center for Research, Economic Advancement, Technology, Engineering, and Science, Albany, NY 12203 USA

**Keywords:** Silicon photonics, Integrated optics

## Abstract

The field of integrated photonics has advanced rapidly due to wafer-scale fabrication, with integrated-photonics platforms and fabrication processes being demonstrated at both infrared and visible wavelengths. However, these demonstrations have primarily focused on fabrication processes on silicon substrates that result in rigid photonic wafers and chips, which limit the potential application spaces. There are many application areas that would benefit from mechanically-flexible integrated-photonics wafers, such as wearable healthcare monitors and pliable displays. Although there have been demonstrations of mechanically-flexible photonics fabrication, they have been limited to fabrication processes on the individual device or chip scale, which limits scalability. In this paper, we propose, develop, and experimentally characterize the first 300-mm wafer-scale platform and fabrication process that results in mechanically-flexible photonic wafers and chips. First, we develop and describe the 300-mm wafer-scale CMOS-compatible flexible platform and fabrication process. Next, we experimentally demonstrate key optical functionality at visible wavelengths, including chip coupling, waveguide routing, and passive devices. Then, we perform a bend-durability study to characterize the mechanical flexibility of the photonic chips, demonstrating bending a single chip 2000 times down to a bend diameter of 0.5 inch with no degradation in the optical performance. Finally, we experimentally characterize polarization-rotation effects induced by bending the flexible photonic chips. This work will enable the field of integrated photonics to advance into new application areas that require flexible photonic chips.

## Introduction

The field of integrated photonics has advanced rapidly due to wafer-scale fabrication, with integrated-photonics platforms and fabrication processes being demonstrated at both infrared wavelengths^1,2^ (with a focus on the driving application areas of LiDAR and communications^3–9^) and visible wavelengths^10–16^ (delving into emerging applications areas, such as displays, optogenetics, and quantum systems^17–24^). However, these demonstrations have primarily focused on fabrication processes on silicon substrates that result in rigid photonic wafers and chips, which limit the potential application spaces.

There are many application areas that would benefit from mechanically-flexible integrated-photonics wafers, such as wearable healthcare monitors that conform to the body or clothing^25–28^ and pliable displays^29,30^. For example, wearable healthcare monitors that would benefit from photonics-enabled functionality are commonly worn on the wrist (as a watch), on the finger (as a ring), or on the upper arm (as a patch); given typical adult wrist circumferences, ring sizes, and upper arm dimensions, these monitors would require bend diameters around 1.6 inch, 0.6 inch, and 3 inches, respectively^31^.

To date, there have been some prior demonstrations of mechanically-flexible photonics fabrication^32–39^. One popular technique that has been used to achieve flexible photonics utilizes heterogeneous integration, where devices are initially fabricated on a rigid substrate and then transferred to a flexible substrate via either a direct-flip process or a stamp-assisted process^33–35^. Another popular technique is monolithic integration, where devices are patterned directly on a flexible substrate that is temporarily supported by a rigid substrate^36–39^. However, these prior flexible photonics demonstrations have been limited to fabrication processes on the individual device or chip scale, which limits scalability.

In this paper, we propose, develop, and experimentally characterize the first 300-mm wafer-scale platform and fabrication process that results in mechanically-flexible photonic wafers and chips. First, we develop and describe the 300-mm wafer-scale CMOS-compatible flexible platform and fabrication process. Next, we experimentally demonstrate key optical functionality at visible wavelengths, including chip coupling, waveguide routing, and passive devices. We experimentally demonstrate fiber-to-chip edge coupling with 8 dB/facet coupling loss, propagation losses for 300-nm-wide and 400-nm-wide waveguides of 12.1 dB/cm and 9.4 dB/cm, respectively, and a splitting ratio of 2.9 dB for 1 × 2 multi-mode interferometer (MMI) splitters, all at an operating wavelength of 632.8 nm. Then, we perform a bend-durability study to characterize the mechanical flexibility of the photonic chips. We demonstrate bending a single flexible photonic chip 2000 times around cylinders with diameters ranging from 2 inches to 0.5 inches, with no noticeable degradation in optical performance. Finally, we experimentally characterize polarization effects induced by bending the flexible photonic chips. We compare device performance of the flexible chip lying flat versus while the chip is bent around two cylinders with varying diameters, and we find that the polarization of the output light changes as the chip is bent. This work paves the way for scalable flexible integrated-photonics fabrication and will enable the field of integrated photonics to advance into new application areas that require flexible photonic chips.

## Results

### Wafer-scale fabrication process

The flexible-wafer platform and CMOS-compatible 300-mm wafer-scale integrated-photonics fabrication process were developed at the New York Center for Research, Economic Advancement, Technology, Engineering, and Science’s (NY CREATES) Albany NanoTech Complex (the facility that houses AIM Photonics). All wafer-level processing was performed using NY CREATES’s suite of advanced 300-mm wafer processes for 193-nm immersion lithography, thin-film chemical vapor deposition (CVD), reactive-ion etch (RIE), chemical-mechanical planarization (CMP), and wet cleaning in Albany, NY.

**Figure 1:**
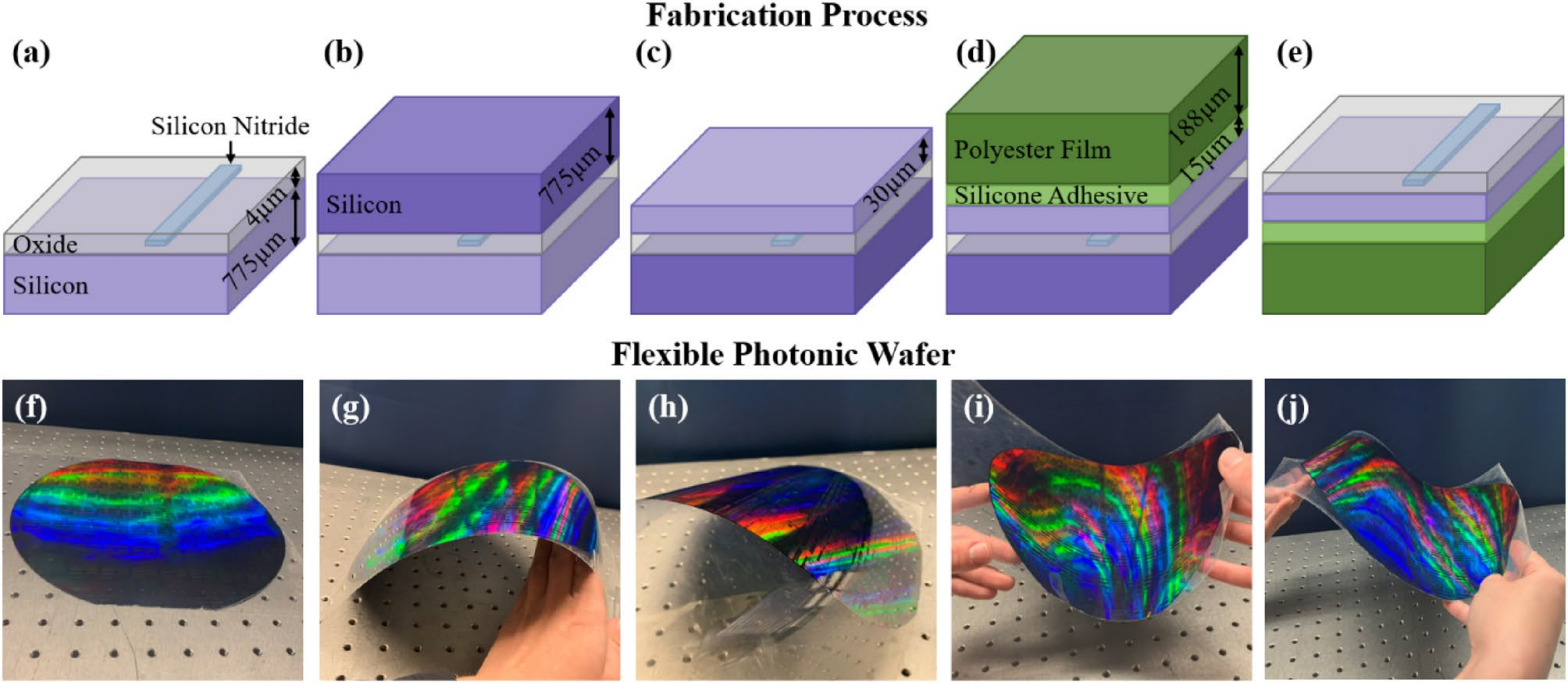
Stack diagrams (not to scale) depicting process flow as (**a**) the silicon-dioxide and silicon-nitride layers are fabricated, (**b**) a temporary silicon handle wafer is bonded on top of the oxide, (**c**) the wafer is flipped and the silicon is thinned down, (**d**) a polyester film is attached on top of the silicon layer, and (**e**) the wafer is flipped and the temporary silicon handle wafer is removed. Photographs of the fabricated flexible integrated-photonics wafer (**f**) flat, (**g**) convexly curved in one direction, (**h**) convexly curved in the other direction, (**i**) concavely curved, and (**j**) curved convexly and concavely simultaneously.

The first part of the process sequence is the fabrication of the photonic devices. These devices were fabricated on a 300-mm-diameter silicon wafer substrate covered with a 2-µm-thick tetraethylorthosilicate (TEOS) plasma enhanced chemical vapor deposition (PECVD) silicon-dioxide (SiO_2_) layer. The thickness of this silicon-dioxide layer was measured to be 2.0 ± 0.1 µm. A silicon-nitride (Si_3_N_4_) layer was then deposited on top of this silicon-dioxide thin film using a PECVD process. The thickness of this silicon-nitride film as deposited was measured to be 180 ± 6 nm. Both the silicon-dioxide and the silicon-nitride layers were deposited on industry-standard 300-mm CVD platforms. This deposited silicon-nitride film was subsequently polished by a chemical-mechanical planarization (CMP) step in an advanced CMP tool, resulting in a smooth surface and a final layer thickness of 160 ± 8 nm. The silicon-nitride layer was then patterned using 193-nm immersion lithography and a dry-etch process on a 300-mm production etch system. As an example, for a designed waveguide width of 300 nm, the fabricated post-lithography width was measured to be 315 ± 5 nm, and the post-etch width was measured to be 300 ± 10 nm at the top of the waveguide with a 2° taper on the waveguide sidewall that resulted in an 11 nm increase in the width at the bottom of the waveguide. Another TEOS PECVD SiO_2_ layer was then deposited on top of the Si_3_N_4_ layer, and its top surface was planarized back using another CMP step, leaving 2 ± 0.1 µm of planarized SiO_2_ over the Si_3_N_4_ layer, as depicted in Fig. 1a. All of these process steps were performed at temperatures below 500 °C in order to minimize wafer bending due to film stress induced by the thermal mismatch between the dielectric layers and the silicon wafer. Minimizing wafer bending was necessary because the subsequent wafer-thinning process (described below) is sensitive to wafer bow. A consequence of this thermal processing constraint was higher waveguide propagation loss for the silicon-nitride waveguides (discussed in detail in the following section).

Once the photonics stack was completed, a standard 300-mm-diameter silicon handle wafer was temporarily bonded to the top surface of the SiO_2_ layer in a 300-mm wafer bonder tool using an adhesive bonding material, as depicted in Fig. 1b. Then, the bonded wafer pair was flipped over, and the original silicon substrate was ground down to 30 ± 10 µm using a combination of course and fine grinding steps on a 300-mm grinder/polisher tool, as shown in Fig. 1c. Next, a 188-µm-thick polyester film with a 15-µm-thick silicone adhesive layer was adhered to the top of the thinned silicon layer, as shown in Fig. 1d. Finally, the wafer was flipped back over, and the temporary silicon handle wafer was removed, as depicted in Fig. 1e. Photographs of the fabricated flexible integrated-photonics wafer are shown in Fig. 1f-j. The photonic wafer was then diced into individual chips in the MIT.nano cleanroom for experimental testing.

### Optical characterization

**Figure 2:**
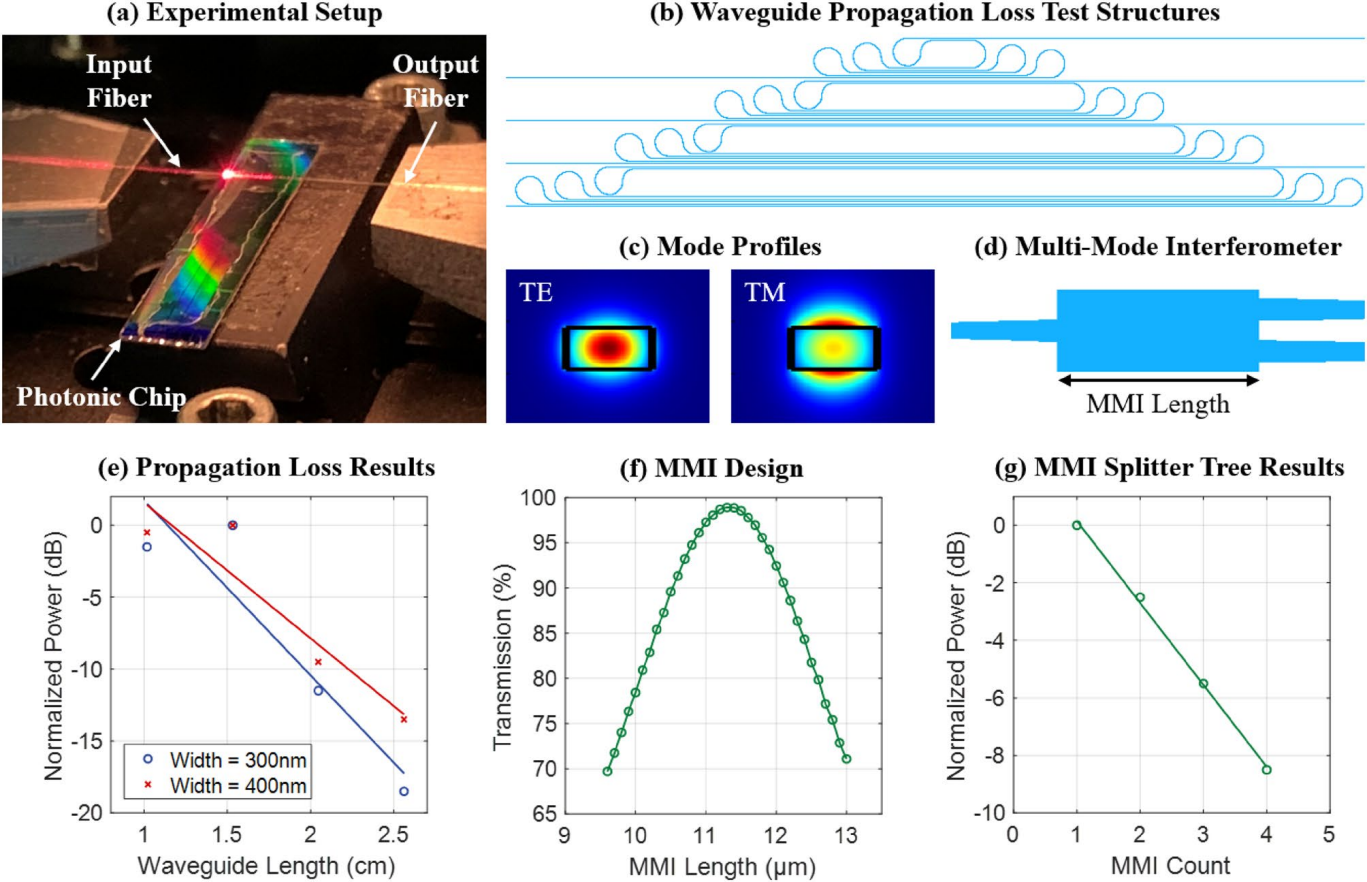
(**a**) Photograph of a diced flexible photonic chip on the experimental setup. (**b**) Top-view diagram of a suite of paperclip loss test structures with four varying waveguide lengths. (**c**) Simulations of the mode profiles for the fundamental transverse-electric (TE) and transverse-magnetic (TM) modes supported by the 400-nm-wide silicon-nitride waveguides. (**d**) Top-view diagram of a 1 × 2 MMI splitter, with the MMI length labeled. (**e**) Experimental results showing the measured normalized output optical power for a suite of paperclip loss test structures with four varying waveguide lengths and two waveguide widths, used to calculate waveguide loss. (**f**) Simulated transmission through the MMI splitter versus MMI length, used to design the MMI splitter. (**g**) Experimental results showing the measured normalized output optical power as a function of number of MMI splitters in a four-stage splitter tree, confirming 2.9-dB splitting.

First, we experimentally demonstrated key optical functionality, including chip coupling, waveguide routing, and passive devices. To experimentally characterize the flexible photonic chips, the output from a 632.8-nm-wavelength helium-neon laser was coupled on the chip via a tapered fiber-to-chip edge coupler and the output light was coupled off chip via another edge coupler to a fiber that was routed to a power meter, as shown in Fig. 2a.

First, the coupling loss of these tapered fiber-to-chip edge couplers was measured using a thru waveguide test structure to be approximately 8 dB/facet, which was limited by the facet roughness and variability introduced during the dicing process. The current fabrication process does not include a wafer-level etched dicing trench, which would enable a smoother facet and lower-loss edge coupling, because the fabrication process requires a flat surface topography for performing the wafer transfer process. This is required to facilitate the adhesive bonding step and the resulting bond integrity. Due to this restriction, the ends of the tapered edge couplers were not cut and smoothed in line; instead, the facet was formed via the chip-dicing process. Process optimization for improved edge coupling will be implemented in a subsequent iteration of this platform.

Next, waveguide loss was experimentally characterized using a suite of paperclip loss test structures, which are comprised of sections of straight waveguides with curves to loop back the waveguide and build up more propagation length. One suite of test structures consists of four individual paperclip test structures with four varying waveguide lengths. The paperclip test structures are designed such that each variant includes the same number of loop-back curves to negate the effect of any curved waveguide loss. A top-view diagram of a suite of paperclip test structures is shown in Fig. 2b. We fabricated and experimentally characterized two suites of paperclip test structures: one with a waveguide width of 300 nm and the second with a waveguide width of 400 nm. These two widths were selected because they do not support higher-order modes (example simulated fundamental transverse-electric and transverse-magnetic mode profiles for the 400-nm-wide waveguide are shown in Fig. 2c). The measured propagation losses for the 300-nm-wide and 400-nm-wide waveguides were 12.1 dB/cm and 9.4 dB/cm, respectively, as shown in Fig. 2e (with some uncertainty due to variability in the edge coupler loss as discussed above). These measured losses are expected given that the waveguide fabrication process did not include processing for line-edge-roughness optimization (we measured similar losses for waveguides fabricated in a traditional process on a silicon handle wafer).

These waveguide propagation losses are higher than values previously reported for flexible photonic chip demonstrations, which report losses on the order of 1 dB/cm^36,37^; however, most importantly, those prior demonstrations utilized either device-scale or chip-scale fabrication processes that greatly limit scalability compared to our wafer-scale process (in addition to operating at infrared wavelengths, utilizing non-CMOS-compatible materials and processes, or requiring large waveguide and device dimensions). When comparing our platform to other state-of-the-art silicon-nitride-based foundry platforms on rigid substrates, there are lower waveguide propagation loss values reported at operating wavelengths in the infrared range, where material absorption coefficients are lower than at visible wavelengths. When comparing to other silicon-nitride-based platforms on rigid substrates that operate at visible wavelengths, propagation losses have been reported on the order of 0.1 to 5 dB/cm^13–16^; these lower loss values are often reported for wide waveguide dimensions that effectively reduce scattering losses caused by waveguide roughness at the cost of supporting higher-order modes, for platforms that are fabricated using techniques that are not compatible with high-throughput manufacturing (such as electron-beam lithography), or for platforms that implement low-pressure-chemical-vapor-deposition (LPCVD) silicon nitride, which can produce higher-quality silicon nitride compared to PECVD silicon nitride, thus lowering propagation loss.

In the past, we have demonstrated that an annealing step applied during the wafer-scale waveguide fabrication process reduced sidewall line-edge-roughness effects and resulted in an approximately 3-dB/cm improvement in the waveguide propagation loss for a traditional process on a silicon handle wafer. This waveguide annealing step was not done during the fabrication process for these flexible photonic wafers, due to wafer bowing concerns during the wafer transfer process (discussed in detail in the previous section). However, in future iterations of this fabrication process, we will explore the feasibility of performing an annealing step to reduce waveguide propagation loss. Additionally, for this initial demonstration, we used PECVD silicon nitride; in the future, we will explore using LPCVD silicon nitride to improve waveguide propagation loss due to material losses. Finally, for applications where reducing propagation loss is critical, wider waveguide widths could be implemented to further reduce waveguide propagation loss due to sidewall scattering.

Finally, to demonstrate a more complex device and characterize chip-scale fabrication variation, a four-stage splitter tree consisting of 1 × 2 multi-mode-interferometer (MMI) splitters was fabricated and experimentally measured. A top-view diagram of the MMI is shown in Fig. 2d. An MMI is a symmetric 1-to-2-waveguide splitter device based on self-imaging principles that is designed to evenly split the input light to two output ports^40^. During the design process, the MMI width was chosen to be 2.8 µm to support a few higher-order modes in the MMI region. Given the selected MMI width, the MMI length was then chosen to optimize transmission to the two output ports. Simulation results showing the transmission into the symmetric output mode versus MMI length is shown in Fig. 2f. A final MMI length of 11.3 µm was chosen to optimize transmission through the device. We then cascaded four of these MMIs together into a four-stage splitter tree test structure. Using this fabricated splitter tree, the splitting ratio for a single MMI was experimentally characterized and calculated to be 2.9 dB, as shown in Fig. 2g, closely matching the ratio expected from simulation.

### Bend-durability characterization

Next, we experimentally performed a bend-durability study to demonstrate the mechanical flexibility of the photonic chips. Specifically, we optically characterized a flexible photonic chip before bending it and then optically characterized the same chip after bending it multiple times around cylinders of varying diameters until we reached the point of mechanical failure.

**Figure 3:**
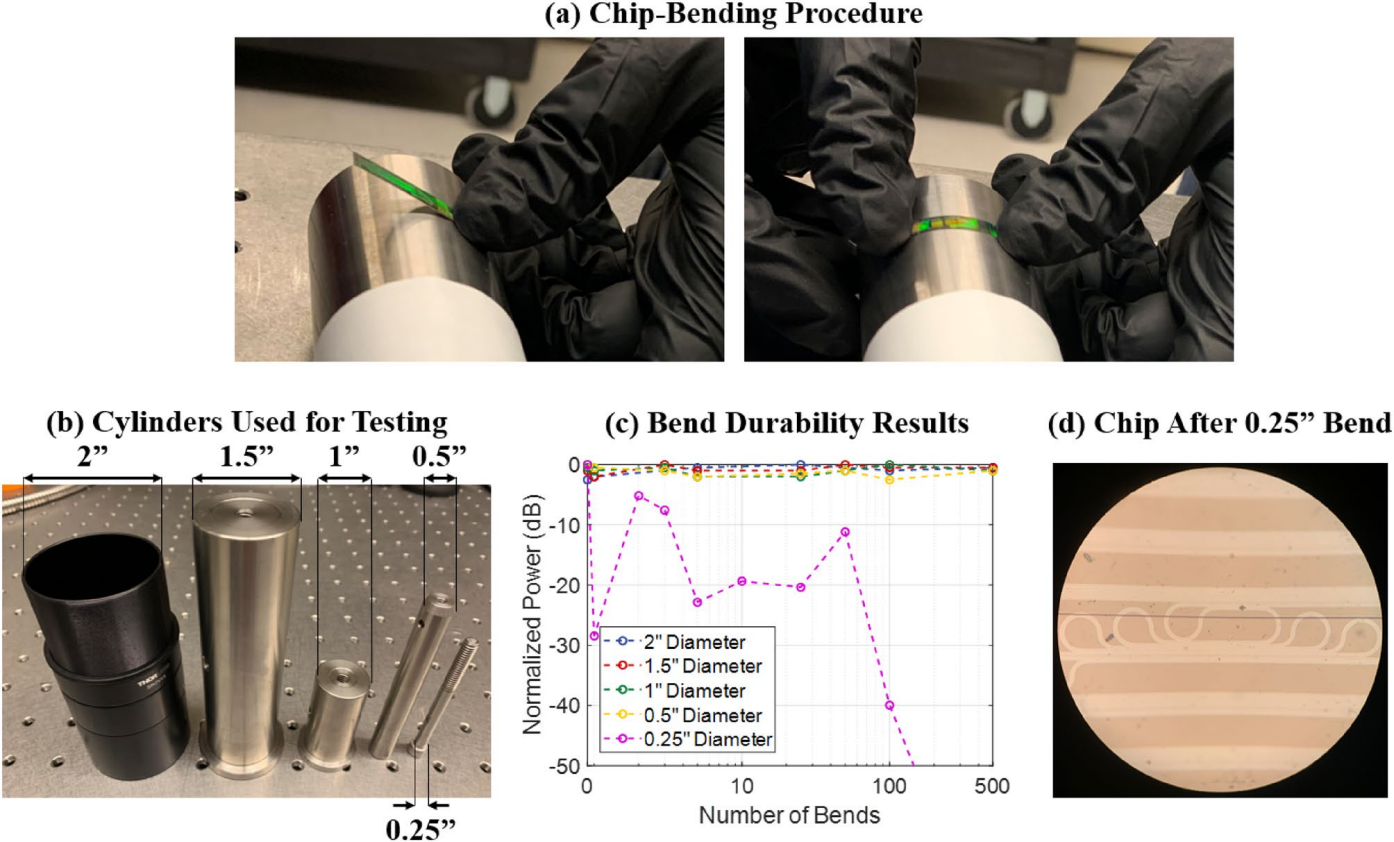
(**a**) Photographs demonstrating the bending procedure for the flexible photonic chip. (**b**) Five cylinders of diameters ranging from 2 inches to 0.25 inches, used during the bend durability testing. (**c**) Experimental results showing the normalized power through the chip versus the number of times the chip was bent around cylinders of varying diameters. (**d**) Micrograph of the photonic chip after it was bent to a diameter of 0.25 inches, showing the failure mechanism of a crack through the waveguide.

As a baseline measurement, first, we measured the optical power through a waveguide test structure while the photonic chip was lying flat on the experimental setup (as shown in Fig. 2a). Then, we took this same chip and bent it around a 2-inch-diameter cylinder. The chip-bending procedure is shown in Fig. 3a; we held one end of the flexible photonic chip against a cylinder and then applied pressure to the other end of the chip to bend it around the circumference of the cylinder. Then, we laid the chip flat on the experimental setup and retested the optical power through the waveguide test structure. We then repeated this process of bending and retesting for an increasing number of bends, up to 500 bends. Next, we took the same chip and repeated this process of bending this single photonic chip around cylinders with diameters of 1.5 inches, 1 inch, 0.5 inch, and 0.25 inch. The cylinders used for this study, with their respective diameters noted, are shown in Fig. 3b.

Experimental results of this study are shown in Fig. 3c. As shown, we were able to bend a single flexible photonic chip 500 times around a 2-inch-diameter cylinder, 500 times around a 1.5-inch-diameter cylinder, 500 times around a 1-inch-diameter cylinder, and 500 times around a 0.5-inch-diameter cylinder, for a total of 2000 bends without any noticeable degradation in the optical performance of the device. Finally, when we bent this flexible chip around a 0.25-inch-diameter cylinder, the chip mechanically failed and the optical power through the device significantly dropped off, as seen in Fig. 3c. Specifically, when the chip was bent around the 0.25-inch-diameter cylinder, a crack formed through the chip that crossed the waveguide test structure, as shown in Fig. 3d. Once the crack formed, the waveguide segments on the two sides of the crack became misaligned. This resulted in reflection and scattering of the light in the waveguide at the interface of the crack, causing a significant reduction in the transmitted optical power (simulation results confirm that even a slight waveguide misalignment of 500 nm in either the horizontal or vertical dimension results in almost no power transmitting through the crack interface).

This bend-durability study exhibits very promising results (on par even with bend-durability results reported for photonics demonstrations fabricated using custom device-scale processes^35^), that the flexible photonic chip can withstand thousands of bends without degradation in optical performance and that device failure does not occur until the chip is bent to an aggressive bend diameter of 0.25 inch (the diameter of a screw). This small of a bend diameter would typically not be necessary for the majority of the application areas relevant for this platform; for example, as discussed above, wearable healthcare monitors would typically need to be bent at diameters ranging from 0.6 inch to greater than 3 inches, which our flexible photonic chips can withstand without degradation in optical performance.

### Bend-induced-polarization-effects characterization

Next, we experimentally characterized the effect of bending the flexible photonic chips on the output polarization. It has been demonstrated that strain can induce a change in the optical properties, such as the birefringence, of a waveguide^41^. When a material is subjected to mechanical distortions, strain can modify the crystal structure, altering the crystal symmetry and polarizability of the material. The resulting change in refractive index of the material is related to this strain by the material’s photoelastic constants. These strain-induced effects can alter the birefringence of the waveguide, resulting in polarization rotation of the light in the waveguide. To characterize these bend-induced polarization effects, we tested a suite of four paperclip loss test structures while a flexible photonic chip was positioned in three different bent configurations, and we observed how the output polarization changes based on the bend diameter.

**Figure 4:**
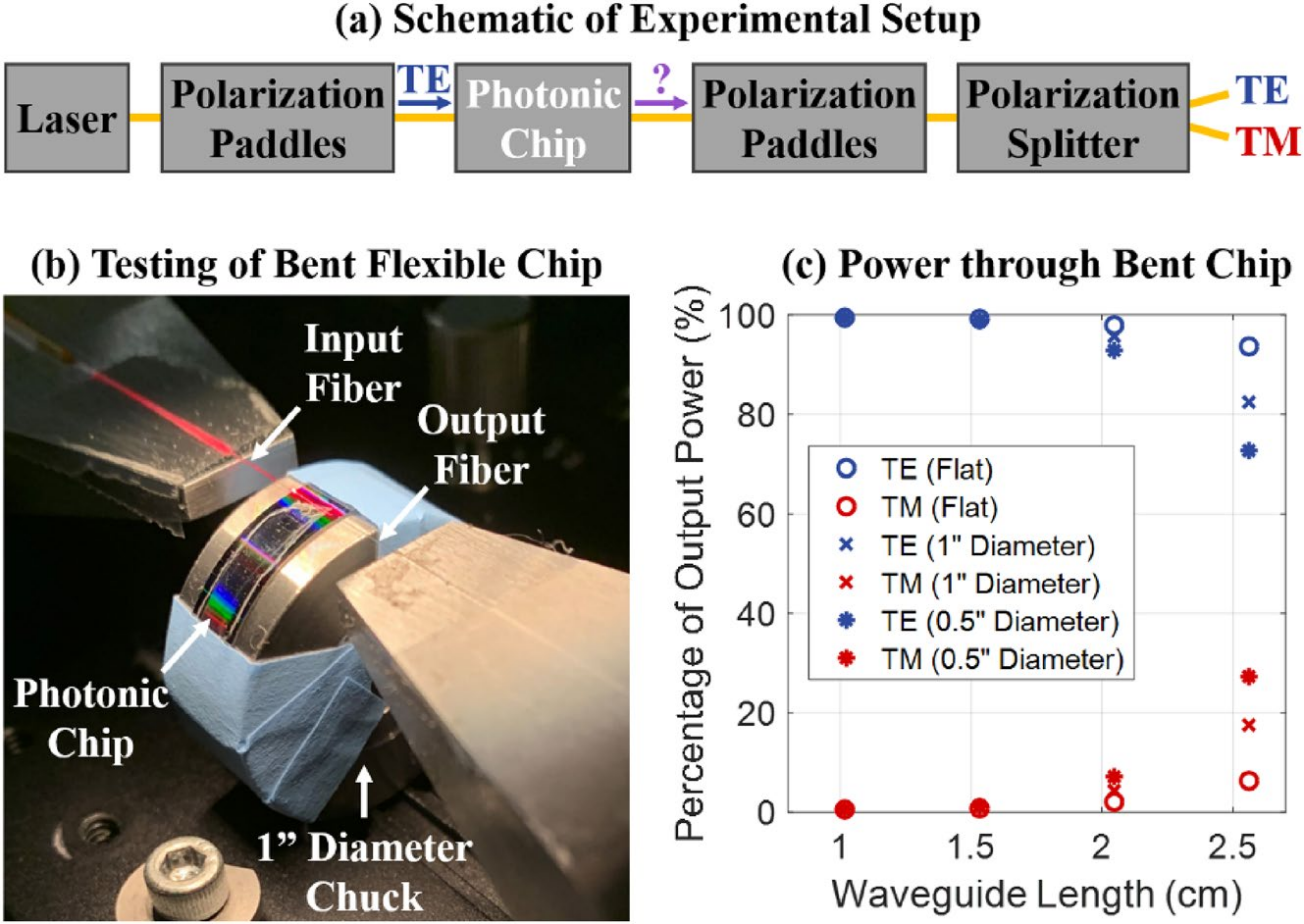
(**a**) Schematic of the experimental setup used to couple TE-polarized light onto the photonic chip and characterize the polarization of the light at the output of the photonic chip. (**b**) Photograph of a flexible photonic chip bent around a 1-inch-diameter cylinder on the experimental setup undergoing optical testing. (**c**) Experimental results showing the percent of the total output power that is TE polarized and TM polarized for a suite of paperclip loss test structures with four varying waveguide lengths, for a flexible photonic chip lying flat, bent around a 1-inch-diameter cylinder, and bent around a 0.5-inch-diameter cylinder.

For this experiment, we coupled transverse-electric (TE) polarized light onto the chip, and we characterized the polarization of the light at the output of the chip for various bend diameters. To set the input polarization, we used a set of polarization paddles. To determine the output polarization, we used a set of polarization paddles and a fiber-based polarization splitter that splits the light into two arms: TE in one arm and transverse magnetic (TM) in the other arm, as depicted in the schematic shown in Fig. 4a. We calibrated the settings of the input and output polarization paddles by using a basic thru test structure on a traditional photonic chip (fabricated in a traditional integrated-photonics process on a rigid silicon handle wafer).

Next, we laid a flexible photonic chip flat on the experimental setup and measured the TE and TM components of the output optical power through the chip for all four paperclip test structures. Then, we took the same flexible photonic chip and bent it around a 1-inch-diameter cylinder on the experimental setup and measured the TE and TM components of the output optical power through the chip for all four paperclip test structures while the chip was bent. A photograph of the flexible photonic chip bent around this 1-inch-diameter cylinder on the experimental setup is shown in Fig. 4b. Finally, we repeated these measurements with the same photonic chip bent around a 0.5-inch-diameter cylinder.

Experimental results of the percent of the total output power that was TE or TM polarized for the four paperclip test structures with increasing waveguide lengths are shown in Fig. 4c. Results are shown for the three bend configurations, namely, the flexible photonic chip laid flat, bent around a 1-inch-diameter cylinder, and bent around a 0.5-inch-diameter cylinder. As the chip was bent to a smaller diameter, the percent of the total output power that was TM polarized increased, suggesting appreciable polarization rotation due to bending for small bend diameters and long waveguide lengths. For example, for the longest length test structure, approximately 6% of the output light was TM polarized when the chip was lying flat, while approximately 27% of the output light was TM polarized when the chip was bent around a 0.5-inch-diameter chuck. In the future, this effect could be leveraged to design a strain sensor that utilizes on-chip polarization detection to sense bend angle, by designing devices with optimized cross sections and lengths that tailor the birefringence between the TE and TM modes to significantly amplify and take advantage of this polarization-rotation effect. On the other hand, for applications where polarization rotation might be unwanted, such as displays, this polarization-rotation effect can be minimized, as long as the system footprint is not too large or the devices are designed to be robust against this polarization rotation.

Moreover, by summing the TE and TM components of the output optical power for the three bend configurations, we also characterized the variation in total output optical power through the chip as a function of the bend radius. As the chip was bent to a smaller diameter, the waveguide propagation loss just slightly decreased for the long waveguide lengths, with approximately 1 dB/cm less loss observed for the 2.5-cm-long test structure when the chip was bent around a 0.5-inch-diameter cylinder compared to when the chip was lying flat. This effect is expected, since, when the chip is bent to a small diameter, some of the input TE light in the waveguide is rotated to the TM mode, and TM modes in integrated waveguides are inherently less lossy compared to TE modes due to reduced interaction with the waveguide sidewalls.

## Discussion

In this paper, we proposed, developed, and experimentally characterized the first 300-mm wafer-scale platform and fabrication process that results in mechanically-flexible photonic wafers and chips.

First, we developed and described the 300-mm wafer-scale CMOS-compatible flexible platform and fabrication process. Next, we experimentally demonstrated key optical functionality at visible wavelengths, including chip coupling, waveguide routing, and passive devices. We experimentally demonstrated fiber-to-chip edge coupling with 8 dB/facet coupling loss, propagation losses for 300-nm-wide and 400-nm-wide waveguides of 12.1 dB/cm and 9.4 dB/cm, respectively, and a splitting ratio of 2.9 dB for 1 × 2 MMI splitters, all at an operating wavelength of 632.8 nm. Then, we performed a bend-durability study to characterize the mechanical flexibility of the photonic chips. We demonstrated bending a single flexible photonic chip 2000 times around cylinders with diameters ranging from 2 inches to 0.5 inches, with no noticeable degradation in optical performance. Finally, we experimentally characterized polarization effects induced by bending the flexible photonic chips. We compared device performance of the flexible chip lying flat versus while the chip was bent around two cylinders with varying diameters, and we found that the polarization of the output light changed as the chip was bent.

In the future, we will both continue developing this wafer-scale fabrication process and perform further in-depth characterization of the resulting flexible photonic chips. First, we will introduce a wafer-level etched dicing trench to the platform to enable a smoother facet and lower-loss edge coupler. Second, we will explore the feasibility of performing an annealing step and switching to an LPCVD silicon nitride to further reduce the waveguide propagation loss. Third, we will perform further in-depth numerical analysis and experimental characterization of the mechanical properties of the flexible photonic chips, including quantifying the Young’s modulus, elastic constants, and fracture strength. Fourth, we will numerically model the impact of strain on the optical properties of the waveguide, and we will compare these simulated results with experimental results for the polarization rotation due to bending. Fifth, we will explore the effects of bending the chip in the other direction, which we expect will have an impact on the polarization rotation due to differences in the stress and strain imparted on the waveguides. Sixth, we will develop a setup to perform automated bend testing to enable rigorous high-throughput characterization of the chip durability. Seventh, we will design more tailored photonic devices to further investigate the effects of chip bending on polarization and loss.

This work paves the way for scalable flexible integrated-photonics fabrication and will enable the field of integrated photonics to advance into new application areas that require flexible photonic chips, including wearable healthcare monitors that conform to the body or textiles and pliable displays^25–30^.

## Data Availability

Data is available upon reasonable request to the corresponding author.
